# Superconducting transition edge bolometer for high-flux neutron detection

**DOI:** 10.1038/s41598-023-49469-4

**Published:** 2023-12-14

**Authors:** Mette Bybjerg Brock, Emil Visby Østergaard, Matteo Busi, Anders C. Wulff, Asger Bech Abrahamsen, Luise Theil Kuhn

**Affiliations:** 1 Department of Energy Conversion and Storage, Technical University of Denmark, 2800 Kgs. Lyngby, Denmark; 2 Laboratory for Neutron Scattering and Imaging, Paul Scherrer Institute, 5232 Villigen, Switzerland; 3 Department of Wind and Energy Systems, Technical University of Denmark, 4000 Roskilde, Denmark; 4 Present Address: Department of Physics, Technical University of Denmark, 2800 Kgs. Lyngby, Denmark; 5 Present Address: SUBRA A/S, 3520 Farum, Denmark

**Keywords:** Materials for devices, Electronics, photonics and device physics, Superconducting devices, Techniques and instrumentation

## Abstract

Needs for neutron detection and monitoring in high neutron flux environments are increasing in several different fields. A completely solid-state, current mode bolometric detector is constructed as a solid substrate transition edge sensor based on a high-T$_c$ superconducting meander. The detector consists of four individual pixels of which three pixels include ${^{10}{\hbox {B}}_{4}\hbox {C}}$ neutron absorption layers. The absorbed energy per neutron absorption reaction is modelled and compared to experimental data. The response of the tested detector is directly correlated to a cold neutron beam with a flux of ${1.8\times 10^{8}}\,{\hbox {n}/{\hbox {cm}}^2/\hbox {s}}$ modulated by a slit. The signal is found to be an order of magnitude higher than the thermal background. The dynamics described by the temporal saturation constants is governed by a modulation frequency less than ${1}\hbox { Hz}$. The thermal response is dynamic and never fully saturates for ${50}\hbox { s}$ exposures. The efficiency for this proof-of-principle design is 1–2%. Possibilities for optimization are identified, that will increase the efficiency to become comparable to existing solid boron-10 detectors. The existing detectors with event-based read-out have limited functionality in high flux environments. The superconducting bolometer described in this work using current-mode readout will pave the way for high flux applications.

## Introduction

There is an increasing need for monitoring of environments with high neutron flux in several fields ranging from commercial nuclear reactors to large scale research facilities. Examples of facilities with increased brightness include the European Spallation Source (ESS) currently under construction^1^, J-PARC^2^, and the Spallation Neutron Source (SNS)^3^. An example within the energy sector is the growing interest in fusion energy, e.g. ITER^4^. Such facilities require neutron detectors to perform plasma diagnostics in extreme flux and environment conditions. These measurements are in parts performed through measuring the flux of high energy neutrons^5,6^.

Superconductors have been used for photon detection for the past 50 years in several research fields^7–9^. The advantages of using superconducting detectors include high sensitivity, high temporal and spatial resolution and a large detection range. For neutron detection, the thermal energy resulting from the most likely neutron absorption reaction (97%) in enriched boron, ${\hbox {n} + ^{10}{\hbox {B}} \longrightarrow ^{7}{\hbox {Li}} + ^{4}{\hbox {He}}}({2.31}\hbox { MeV})$ is very small. The high sensitivity of a superconducting bolometer would provide a method to detect this energy directly, thus avoiding additional amplification steps.

Existing superconducting detectors for neutron detection are relatively few and so far non-commercial. Merlo et al.^10^ have previously tested a superconducting neutron sensitive bolometer consisting of a single Nb strip with a width of ${10}\hbox { nm}$. The Nb-strip detector has a dead time of 120s. This is far from the state-of-the-art of ${\upmu \hbox {s}}$ for ${^{3}\hbox {He}}$ gas detectors^11^, and ${100}\hbox { ns}$ for the recent Gas Electron Multiplier (GEM) based detector^12^. For ${^{6}\hbox {LiF:ZnS(Ag)}}$ scintillator detectors the dead time is in the range of at least tens of ${\upmu \hbox {s}}$ and the long fluorescence decay can lead to count rate limitations^13,14^. Using arrival time statistics this can be reduced to ${3}\,{\upmu \hbox {s}}$^15^. The dead time limits the count rate capability, which is an important feature for high neutron flux environments. The most recent successful results for superconducting neutron detectors have been shown by the Current Biased Kinetic Inductance Detector (CB-KID), which is developed as a neutron imaging system and have dead times on the order of ${\upmu \hbox {s}}$^16–18^. For both superconducting detectors, enriched ${^{10}\hbox {B}}$ was used as the neutron conversion layer.

Enriched boron carbide $({^{10}{\hbox {B}}_{4}\hbox {C}})$ films have been developed for neutron detection at the ESS^19^, where they are used in detectors with an ionizing gas and high-voltage wire read-out. One of these detectors, the boron Multi-Blade, designed for high intensity neutron reflectometry has a dead time of approximately ${1.5}\,{\upmu \hbox {s}}$^20^. The efficiency of a ${4}\,{\upmu \hbox {m}}$ single layer is maximum 6% at a neutron wavelength of 2.5 Å, for incident neutrons normal to the absorption plane^21^. Several layers can be used to obtain a higher efficiency, which has been shown in the concept of the Multi-Grid detector. This detector, that was designed for the ESS spectrometers, reached an efficiency above 65% at 3.5 Å for 32 layers of 0.5–${1.5}\,{\upmu \hbox {m}}$ thick ${^{10}{\hbox {B}}_{4}\hbox {C}}$^22^. Alternatively, a grazing angle geometry is adopted to increase efficiency up to 60% at ${5}^{\circ }$ incident angle of 3.5 Å neutrons using the Multi-Blade detector^23^.

Enriched boron carbide based, current-mode, no-gain detectors have recently been developed for monitoring in specific experimental areas close to the source at ESS. These detectors are still under optimization to be suitable for the operation in a high neutron flux environment ($>{10^{5}}{\hbox {n}/{\hbox {cm}}^{2}/\hbox {s}}$) in the vicinity of the neutron source^24^.

We present here a solid-state superconducting transition edge bolometer (TEB). The bolometer consists of a superconducting meander-shaped pixel with an enriched boron carbide $({^{10}{\hbox {B}}_{4}\hbox {C}})$ neutron absorption layer deposited on top. The detector was tested at the BOA beamline^25^, at the Swiss Spallation Neutron Source (SINQ)^26^, at the Paul Scherrer Institute (PSI), to evaluate its performance as a neutron detector.

## Methods

### Detector design

The detector consists of four pixels, each with a meander pattern made of Yttrium Barium Copper Oxide (YBCO) with two electrodes covered by a silver (Ag) top layer. YBCO was chosen as the high temperature superconductor (HTS) material for this detector, due to the ease of fabrication into thin films, moreover it is a well-known material^27^. It has a high critical temperature, $T_c$, which can be tuned by oxygen depletion or doping. This allows it to be adapted to different operational requirements. The critical current of YBCO has a very slow degradation rate with increasing magnetic field, which enables YBCO-based detectors to operate in environments of high magnetic fields on the order of tens of Tesla.

**Figure 1:**
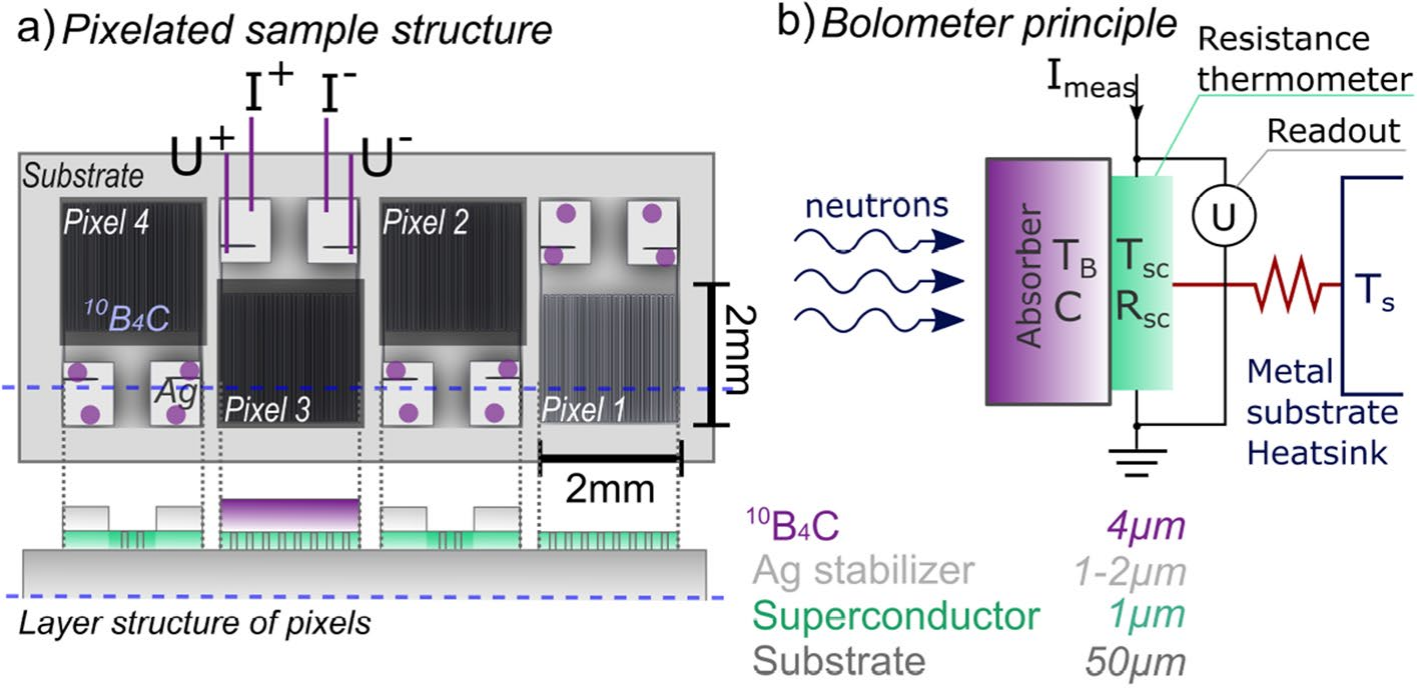
(**a**) Drawing of the detector with four meander-shaped pixels, where pixels 2–4 are coated with enriched ${^{10}{\hbox {B}}_{4}\hbox {C}}$. Measuring points for the four-point measurements are marked by circles. A cross-section drawing of the layer structure is indicated by the dashed blue line and is shown at the bottom of the figure. (**b**) Principle of the bolometric detection of neutrons with an absorption layer combined with a transition edge bolometer (TEB). The detector is comprised of a solid stack and the thermal bridge is therefore defined by the interface resistances between layers in the stack.

The pixel meanders of the prototype have an active area of 2$\times {2}\,{{\hbox {mm}}^2}$ with ${50}\,{\upmu \hbox {m}}$ path widths and a fill factor of 0.5. The fill factor refers to the ratio of active area paths and the passive gap between them. The meander shapes are chosen to maximize the ohmic resistance by creating a long and thin current path. An increased normal-state resistance leads to a steeper transition to the superconducting state at the critical temperature ($T_c$). The large change in resistance over a small temperature range is the principle behind the superconducting thermometer. A drawing of the detector setup is shown in Fig. 1a.

Three of the four detector pixels are coated with a ${4}\,{\upmu \hbox {m}}$ thick ${^{10}{\hbox {B}}_{4}\hbox {C}}$ neutron conversion layer, for neutron detection capability, as depicted by the dark squares covering the meanders in Fig. 1a. Pixel 1 was not coated, and thereby not sensitive to neutrons. This design was chosen to measure the background signal from other types of radiation, e.g. gamma, or from other thermal sources.

The ${^{10}{\hbox {B}}_{4}{\hbox {C}}}$ was deposited by DC magnetron sputtering at the ESS Detector Coatings Workshop. The process is similar to the one described in a previous paper^28^.

The average boron-10 neutron absorption reaction releases an energy of ${2.79}\,{\hbox {MeV}}$^29^, converted into heat and transferred to the TEB underneath. The TEB pixel meanders are positioned on a metallic substrate, which acts as a heat sink. The heat dissipated by the neutrons in the absorption layer is transported through the superconducting meander structure into the substrate. A principle diagram of the bolometer detector is shown in Fig. 1b. The main components are the conversion layer, the superconducting thermometer, and the connection to the heat sink (metallic substrate material).

The pixel and the metallic substrate structure was fastened to a customized, ceramic, aluminum nitride (AlN), printed circuit board (PCB). The electrical connections to the PCB were made using gold (Au)-wirebonds. Electrical contacts between the PCB and the read-out system were made by soldering thermally compensated wires onto the PCB.

### Cryogenic and electronic measuring setup

**Figure 2:**
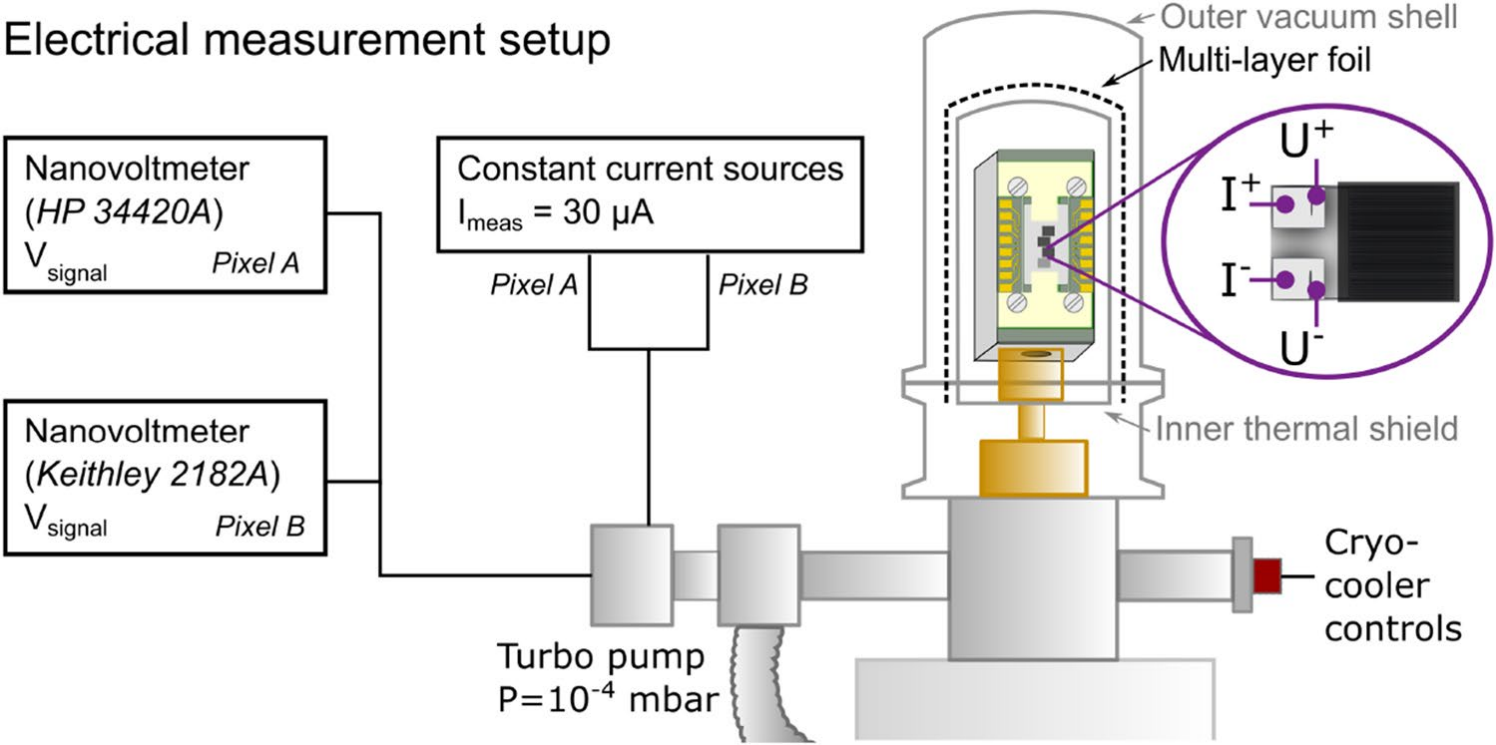
Diagram of the cryogenic system. The Al frame holding the detector and PCB is connected to the cold head of the cryocooler with Cu parts. The inner and outer thermal radiation shield consists of solid Al cylinders with multi-layer insulation foils in between. The detector environment is pumped to a vacuum of ${10^{-4}}\,{\hbox {mbar}}$ to limit convective heat transfer. The enlarged pixel shows the position of the four probes used to perform the 4-point voltage measurements. The equipment used to provide the measurement current ($I_{{\textrm{meas}}}$) and measure the voltage of each pixel is shown (black boxes).

The test detector was mounted on an aluminum (Al) block. The block was attached to the cold head of an AIM SL400 linear Stirling cryocooler using copper (Cu) connectors as it is shown in Fig. 2. The detector and wires were enclosed by an Al cap allowing for a vacuum of ${10^{-4}}\,{\hbox {mbar}}$. The thermal shielding consisted of: (i) an outer Al cylinder co-functioning as vacuum bell (shell thickness ${1}\,{\hbox {mm}}$), (ii) a multi-layer, Al-based, insulation foil kept in place with Al tape, and (iii) an inner Al cylinder (shell thickness ${0.4}\,{\hbox {mm}}$). The inner Al shell was connected to the cold head. The thermal shielding consisted mainly of Al as this material is highly transparent to neutrons. The cryocooler set-point temperature was stable within $\pm {10}\,{\hbox {mK}}$.

The bias current to the detector was supplied by two adjustable LakeShore 120 current sources, while the voltage signal was measured using HP 34420A and Keithley 2182A nanovoltmeters. The sampling rate was approximately ${8}\,\hbox {Hz}$ based on the integration time of the nanovoltmeters of approximately ${120}\,{\hbox {ms}}$. The bias current for each pixel was ${30}\,{\upmu \hbox {A}}$, which allowed for the largest signal-to-noise ratio, without the risk of thermal runaway by joule heating. The runaway current was calculated to be minimum ${400}\,{\upmu \hbox {A}}$^30^. The measurements were performed as four-point measurements with separate current and voltage leads as shown in Fig. 2.

### Neutron beamline setup

**Figure 3:**
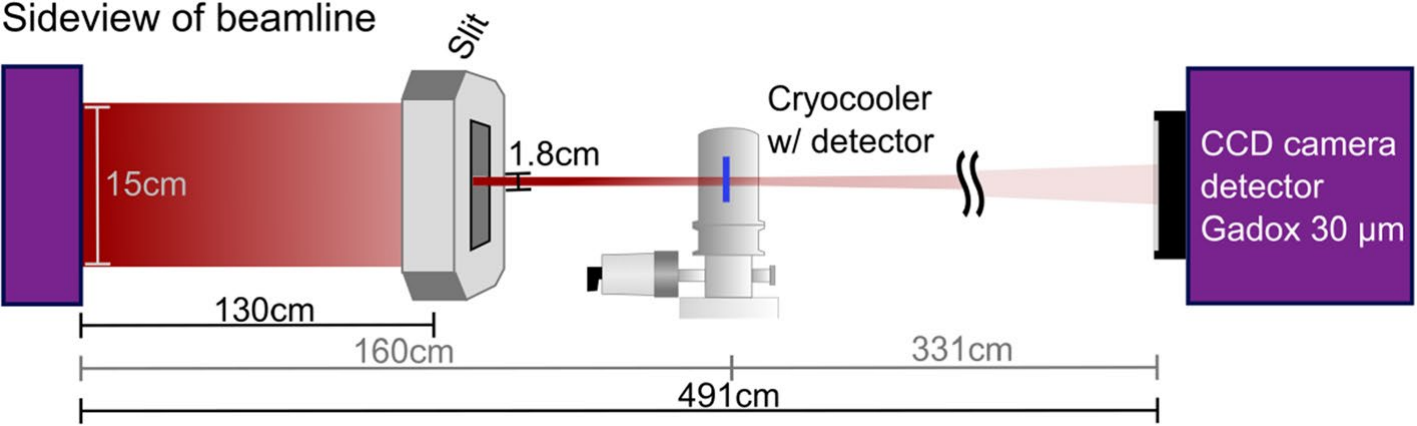
Test setup used for neutron response tests at the BOA beamline at PSI. A slit was used to modulate the neutron beam. A reference CCD camera detector with a ${30}\,{\upmu \hbox {m}}$ Gadox scintillator was placed ${331}\,{\hbox {cm}}$ behind the test detector.

The neutron response tests were carried out at the BOA beamline at the Paul Scherrer Institute (PSI). The beamline has a cold neutron spectrum with peak wavelength of 3.5 Å, corresponding to a peak energy of ${6.7}\,\hbox {meV}$ of the white neutron beam. A flux of ${1.8\times 10^{8}}\,{\hbox {n}/{\hbox {cm}}^2/\hbox {s}}$ was measured at the point where the beam enters the beamline in the cave^25^. The setup used for neutron response tests is shown in Fig. 3 and included a motorized slit mounted in front of the test detector. A CCD reference detector with a ${30}\,{\upmu \hbox {m}}$ Gadox scintillator screen was placed ${331}\,{\hbox {cm}}$ behind the test detector. This position was the closest possible position. No static aperture was used at the beam entrance to the cave. The beam size was modulated by the slit with a maximum vertical and horizontal opening of ${1.8}\,{\hbox {cm}}$ and ${7}\,{\hbox {cm}}$ respectively.

The expected neutron intensity at the position of the test detector was estimated using three Au-foil activation measurements performed previously at the beamline^25^. These were fitted to find the decay function for the flux at a given position along the direct line-of-sight of the beam entrance. This yields an expected flux of ${1.2\times 10^{8}}\,{\hbox {n}/{\hbox {cm}}^2/\hbox {s}}$ at the test detector position, which is equal to ${4.8\times 10^{6}}\,{\hbox {n}/\hbox {s}}$ on each ${4}\,{{\hbox {mm}}^2}$ sized pixel. Response tests consisted of repeated cycles where the slit was first in an open position for time $t_p$, and then in a closed position for time $t_p$. These cycles were repeated for $t_p=20$s, 30s and 50s. Due to the mechanical movement of the slit, the change between open and closed states were not immediate.

Transmission contrast radiographs were acquired at the same beamline without the slit and with apertures of 20$\times {20}\,{{\hbox {mm}}^2}$ and 10$\times {10}\,{\hbox {mm}^2}$ placed at the beam entrance. The detector prototype was placed directly in front of the Gadox scintillator of the CCD reference detector (detector distance $<{2}\,{\hbox {cm}}$).

## Results

### Superconducting transition

**Figure 4:**
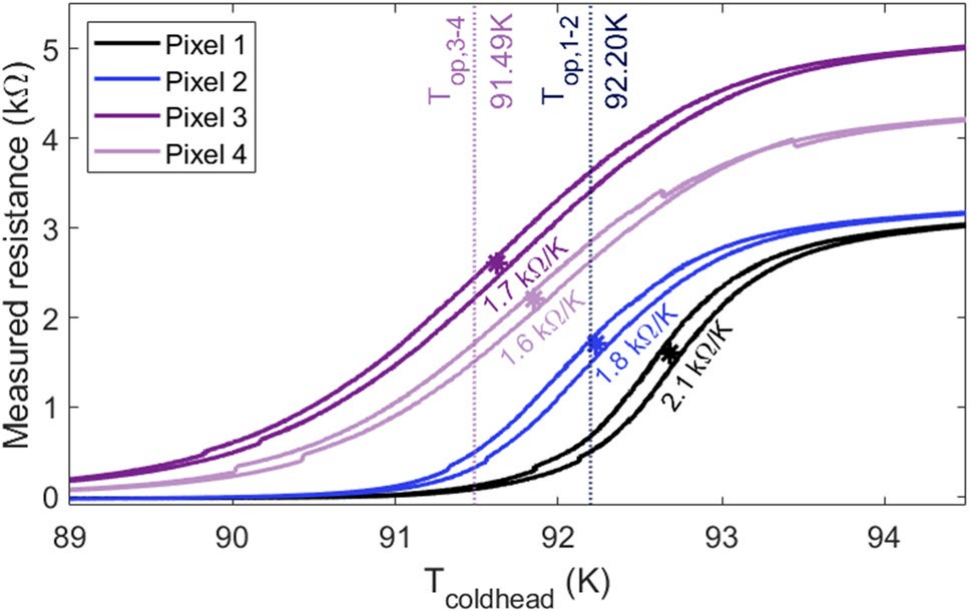
Measured resistance while performing a temperature ramp at a rate of ${0.05}\,{\hbox {K}/\hbox {min}}$. The maximum slope for each pixel is denoted by the star symbol and the corresponding *dR*/*dT* value at the given point is noted. Dashed vertical lines indicate the operating temperatures, $T_{{\textrm{op}}}$, at which the neutron response measurements of pixel 1 and 2 ($T_{{\mathrm {op,1-2}}}$), and pixel 3 and 4 ($T_{{\mathrm {op,3-4}}}$) were performed.

The resistance of the superconducting meander pixels was measured by ramping temperatures at a rate of ${0.05}\,{\hbox {K}/{\hbox {min}}}$ in an interval of $T_c\pm {3}\,\hbox {K}$, where $T_c$ is the expected critical temperature for each of the 4 pixels. Resistance-temperature curves for all four pixels are shown in Fig. 4. The temperature is measured at the position of the cold head. Therefore, the relative working temperature of the detector is recorded, rather than given as an intrinsic value of $T_c$ for each pixel. The offset between the measured temperature and the expected value of $T_c$ was found to be close to ${1}\,\hbox {K}$ and to be constant for a constant cooling or heating rate. The temperature was measured using a surface mounted Si-diode detector placed next to the test detector on the Al block.

The transition for pixels 2 to 4 occurs at a cold head temperature range between 91.63 and ${92.24}\,\hbox {K}$, and at ${92.68}\,\hbox {K}$ for pixel 1, which is the only pixel not coated with a conversion layer. This is in agreement with a previous publication, where the effect of depositing a ${\upmu \hbox {m}}$ thick layer of enriched boron carbide on a high-temperature superconducting meander was investigated. It was reported that the deposition process lowered the transition temperature of the superconductors by ${1.4}\,\hbox {K}$ on average^28^. The apparent hysteresis is caused by a temperature delay in the setup for the cooling compared to the heating procedure and is therefore not a property of the superconductor. The delay was larger than the rate of ${0.05}\,{\hbox {K}/\hbox {min}}$.

The sensitivity of the pixels is directly correlated to the tangent at the operation temperature $T_{{\textrm{op}}}$. The *dR*/*dT* value is found as the maximum of the first order derivative of the logistic function fitted to the measured transition curve. The *dR*/*dT* values for the pixels are in the range of 1591–2111 $\pm {7}\,{\Omega /\hbox {K}}$. The highest value is obtained for the non-coated pixel. This result is in agreement with previous measurements that showed a decrease in sensitivity of 15% to 60% for coated pixels compared to non-coated pixels^28^. The difference in the resistance values measured above $T_c$ is expected to be caused by variations in the fabrication of the individual pixels. Inhomogeneities in the wet etching process results in minor variations in the path-widths of the superconducting meanders.

The reported transition temperatures shown in Fig. 4 were used to regulate the temperature controller so the test detector was operated at temperatures with the best possible sensitivity. Measurements using pixel 1 and 2 were performed at ${92.20}\,\hbox {K}$, while measurements using pixel 3 and 4 were performed at ${91.49}\,\hbox {K}$.

### Analytical model of detector efficiency

The absorption reactions of neutrons in the enriched boron carbide layers are

1 $$\begin{aligned}{}&(94\%) \hspace{2mm} ^{10}\text {B}+n\rightarrow ^7\text {Li}({0.84}\,\hbox {MeV})+^4\text {He}({1.47}\,\hbox {MeV})+\gamma ({0.48}\,\hbox {MeV}) \end{aligned}$$

2 $$\begin{aligned}{}&(6\%)\hspace{2mm} ^{10}\text {B}+n \rightarrow ^7\text {Li}({1.02}\,\hbox {MeV})+^4\text {He}({1.78}\,\hbox {MeV}). \end{aligned}$$

The energies stated in the equations are in the form of kinetic energies of the reaction products. To achieve the highest possible signal for each neutron, all the reaction products should be completely stopped by the absorption layer. For the ion products of ${^{7}\hbox {Li}}$ and ${^{4}\hbox {He}}$ the mean free paths are a few ${\upmu \hbox {m}}$ in enriched boron carbide. Gamma rays have a half value thickness of several cm due to the small absorption cross section of boron carbide^31^. Therefore, it is not expected to be possible to capture gamma rays with this setup, and the thickness of the conversion layer has been optimized solely for the absorption of neutrons and ions from the neutron absorption reaction.

The majority of the available literature on modelling the efficiency of solid boron layers for detection purposes are related to either semiconductor detectors or combined gas-solid detectors. Both detection methods require the charged reaction products to exit the absorption layer to ionize either a gas or a semiconductor volume. With a bolometer detector the aim is to convert all the kinetic energy of the products to thermal energy inside the solid conversion layer to be transported to the superconducting thermometer.

We present here a simplified physical model to describe the efficiency of the TEB for a conversion layer thickness of ${4}\,{\upmu \hbox {m}}$. The model used in this work is based directly on the principles behind a previous model created for boron carbide diode detectors. All equations, assumptions and in-depth descriptions of the methodology can be found in the work by Lundstedt et al.^32^.

**Figure 5:**
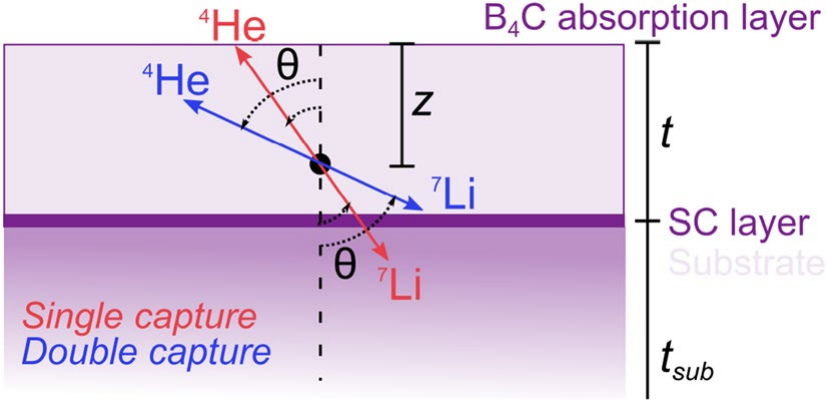
Principle sketch of a neutron capture in a ${^{10}{\hbox {B}}_{4}\hbox {C}}$ conversion layer at depth *z*. The absorption layer has thickness *t*, which is much thinner than the substrate, $t \ll t_{sub}$. The reaction products are ${^{4}{\hbox {He}}}$ and ${^{7}{\hbox {Li}}}$ ions moving at an angle, $\theta$, with regards to the vertical direction. The products are assumed to always move in opposite directions.

A principle sketch of a neutron absorption reaction is depicted in Fig. 5. The absorption results in two ion products (${^{4}{\hbox {He}}}$ and ${^{7}{\hbox {Li}}}$) moving in opposite directions.

The model uses a GEANT4 parametrisation of the Bethe–Bloch equation to describe the energy deposited by the ion products from the absorption reaction in the enriched boron carbide layer ($E_d$).

3 $$\begin{aligned} E_d = \frac{S\cdot z}{\cos \theta } \hspace{3mm}\text {with}\hspace{3mm} S=\frac{E_{{\textrm{ion}}}}{R_{{\textrm{MFP}}}}, \end{aligned}$$

where $E_{{\textrm{d}}}$ is the deposited energy, *S* are the stopping factors in enriched boron carbide of the specific ions, $E_{{\textrm{ion}}}$ are the energies of the ions given by the absorption reactions in Eqs. (1) and (2), and $R_{{\textrm{MFP}}}$ are the mean free paths of the ions in enriched boron carbide. $R_{{\textrm{MFP}}}$ values are calculated using the SRIM software^33^ and the corresponding material parameters. The depth (*z*) and angle of the products ($\theta$) are shown in Fig. 5.

The total energy $E_{{\textrm{T}}}$ for an absorption reaction is determined as the difference between the maximum energy $E_{{\textrm{max}}}$ of the ions and the remaining energy any ion leaving the layer has. This is described by the following relation,

4 $$\begin{aligned} \begin{aligned} E_{{\textrm{T}}}&= E_{{\textrm{max}}}-E_{{\textrm{loss,1}}}-E_{{\textrm{loss,2}}}\\&= (E_{{\textrm{ion,1}}}+E_{{\textrm{ion,2}}})-(E_{{\textrm{ion,1}}}-E_{{\textrm{d,1}}})-(E_{{\textrm{ion,1}}}-E_{{\textrm{d,2}}})\\&= E_{{\textrm{d,1}}}+E_{{\textrm{d,2}}}. \end{aligned} \end{aligned}$$

$E_{{\textrm{ion,i}}}$ is the full kinetic energy of a single ion described in Eqs. (1) and (2). $E_{{\textrm{loss}}}$ describes the remaining energy of the ions travelling outside of the absorption layer, which is then not converted to heat inside the detector. To describe this a piece wise function is applied:

5 $$\begin{aligned} E_{{\textrm{d}}}= {\left\{ \begin{array}{ll} \frac{S\cdot z}{\cos \theta } &{} \text {for} \hspace{4mm} \frac{z}{\cos \theta }\ge R_{{\textrm{MFP,i}}} \\ E_{{\textrm{ion}}} &{} \text {for} \hspace{4mm} \frac{z}{\cos \theta } < R_{{\textrm{MFP,i}}} \end{array}\right. } \end{aligned}$$

Equation (5) is true for all ions travelling upwards in the layer as it is shown geometrically in Fig. 5. For any ion travelling down through the layer, the depth travelled is defined by: $t-z$. Therefore, the boundary of the piece wise function becomes $(t-z)/\cos \theta$ and the energy becomes $E_d=(t-z)\cdot S/\cos \theta$ for these ions.

Each ion has two different energies depending on the absorption reactions (Eqs. (1) and (2)) and each of these energies are defined by a specific $R_{MFP,i}$ and $S_{i}$. Therefore, the calculation of $E_{d,i}$ must be performed separately for each ion in each reaction. To obtain the full spectrum, the energy at every angle $\theta$ and at every absorption depth *z* is summed and normalized. The calculation is performed numerically by stepping over a number of angles from 0 to $\cos \theta =1$ using step-size $\Delta \cos \theta ={5\times 10^{-4}}$, and a number of depths from 0 to $z=t$ using step-size $\Delta z={1}\,{\hbox {nm}}$. The counts at each energy is normalized with the number of steps, $\max (\cos \theta )/\Delta \cos \theta =2000$ and $\max (z)/\Delta z=4000$.

The metallic substrate is much thicker $t_{sub}={50}\,{\upmu \hbox {m}}$ than the enriched boron carbide conversion layer $t={4}\,{\upmu \hbox {m}}$. The substrate consists of heavier atoms than the conversion layer and have shorter ion stopping ranges. Therefore, it is assumed that any ion leaving the absorption layer downwards into the substrate will be absorbed here.

**Figure 6:**
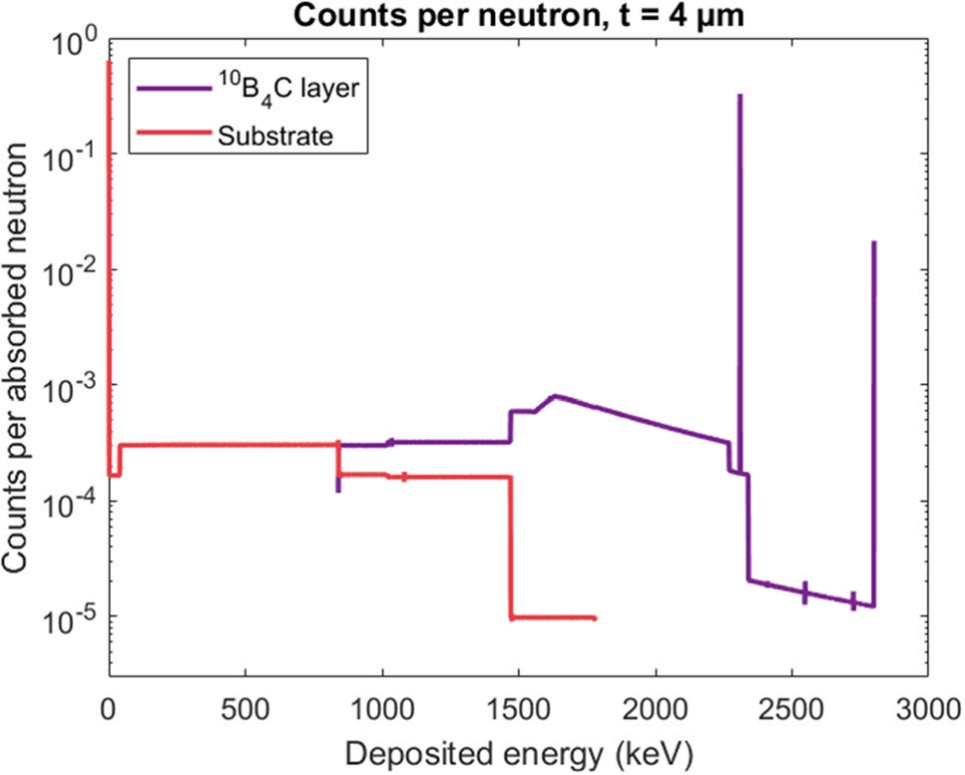
Energy deposition spectrum for a neutron in a ${4}\,{\upmu \hbox {m}}$ thick ${^{10}{\hbox {B}}_{4}\hbox {C}}$ conversion layer (purple curve) and in the substrate (red curve). Calculations based on Eqs. (3)–(5).

Figure 6 shows the energy deposition spectrum calculated for the ${^{10}{\hbox {B}}_{4}\hbox {C}}$ conversion layer (purple curve) and the substrate (red curve). The spectrum in Fig. 6 shows two significant peaks for the absorption in the enriched boron carbide layer at ${2310}\,{\hbox {keV}}$ and ${2800}\,{\hbox {keV}}$. These energies are equal to the full absorption energy of the 94$\%$ and 6$\%$ absorption reactions, respectively. The lowest possible energy deposited in the absorption layer is given by the lowest ion energy in the 94$\%$ absorption reaction, ${^{7}{\hbox {Li}}}$(${0.84}\,{\hbox {MeV}}$). The lower limit is caused by the thickness of the absorption layer being larger than any of the individual mean free paths of the ions. Therefore, any reaction will result in the absorption of the full energy of at least one of the ions. A step is visible for the ${^{4}{\hbox {He}}}$(${1.47}\,\hbox {MeV}$) ion at the full kinetic energy.

The highest possible energy absorbed in the substrate is the full energy of the ${^{4}{\hbox {He}}}$(${1.47}\,{\hbox {MeV}}$) ion. The thickness of the conversion layer only allows a maximum of one of the ions to fully escape to the substrate with the maximum energy ($E_{{\textrm{max}}}$) intact. Therefore, the maximum energy peak is given by the highest $E_{{\textrm{max}}}$ of the four ions. A step can be observed at ${^{7}\hbox {Li}}$(${0.84}\,{\hbox {MeV}}$), which is the minimum full particle energy deposited in the substrate. This minimum energy is determined by the assumption that the absorption reaction takes place in the conversion layer.

The average deposited energy per incident neutron is found to be ${1.877}\,{\hbox {MeV}}$ for the enriched boron carbide layer and ${231}\,{\hbox {keV}}$ for the substrate underneath. A maximum conversion efficiency of $\varepsilon _{{\textrm{conv}}}=\frac{E_{{\textrm{T}}}}{E_{{\textrm{max}}}}=81\%$ can be expected for the direct signal of a detector with ${4}\,{\upmu \hbox {m}}$ enriched boron carbide layer. As the energy deposited in the substrate will be deposited in the first few ${\upmu \hbox {m}}$ of the substrate it might also contribute to the measured signal. Using this assumption yields a maximum efficiency of $\varepsilon _{{\textrm{conv}}}=\frac{E_{{\textrm{T,sub}}}}{E_{{\textrm{max}}}}=91\%$.

### Neutron absorption test

The absorption of neutrons in the enriched boron carbide conversion layer was measured using neutron transmission imaging.

**Figure 7:**
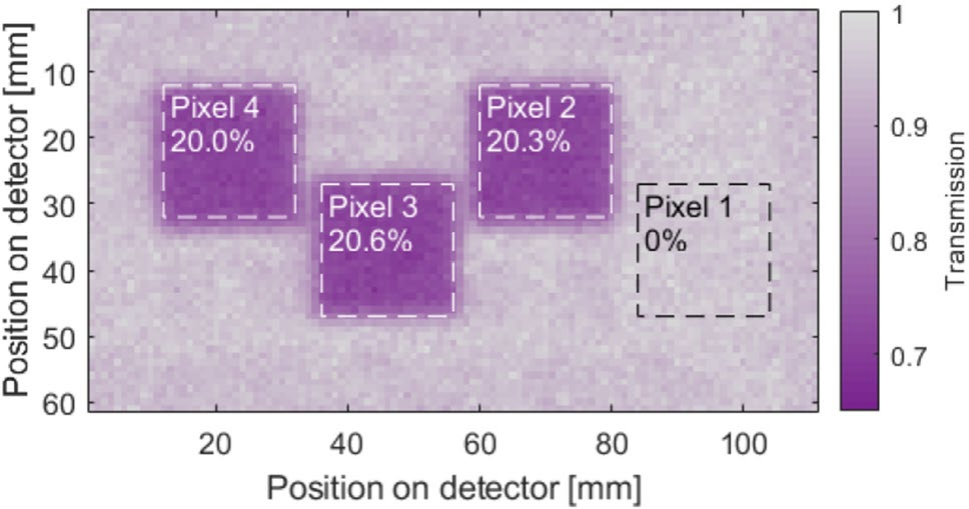
Neutron transmission image of the pixels of the bolometer detector. Areas marked by dotted lines are the pixel areas ($A_{{\textrm{pix}}}$), which have been used to calculate the average absorption compared to the baseline of the surrounding area. Pixel 1 is not coated with enriched boron carbide, and therefore does not absorb any significant number of neutrons.

Figure 7 depicts a neutron transmission image of the four pixels of the detector. The transmission image is an average of five consecutive radiographs each with an acquisition time of ${30}\,\hbox {s}$. Dark current has been subtracted, and the neutron imaging data has been normalized with regards to open beam data acquired before and after the neutron radiography.

No detectable neutron absorption takes place at the position of pixel 1 as shown in Fig. 7. The average neutron absorption of the ${^{10}{\hbox {B}}_{4}\hbox {C}}$-coated pixels 2–4 is found to be 20%.

### Neutron response tests

**Figure 8:**
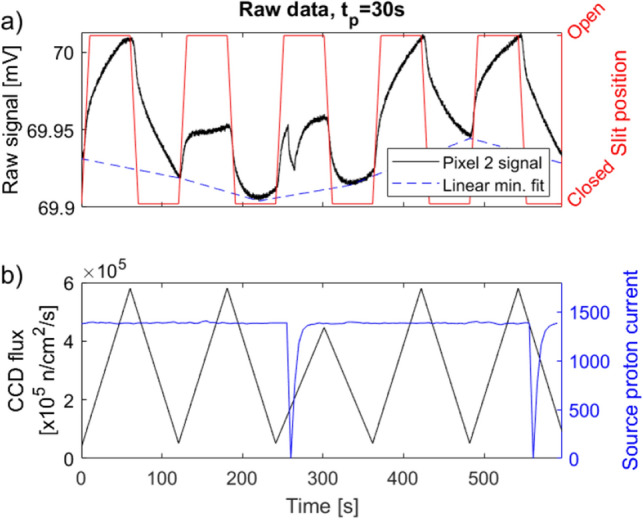
(**a**) Raw voltage signal measured for pixel 2 (black) during exposure to a modulated neutron beam. The neutron beam was modulated using a slit, so an open slit position corresponds to a full beam intensity (red). Linear fits made between each local minima are used to subtract the varying temperature background (dashed blue line). (**b**) Integrated and normalized neutron flux as measured by the reference Gadox-CCD detector (black). The measurement is recorded at the end of each open or closed period. The proton current of the SINQ spallation source creating a neutron flux proportional to the proton current (blue).

The response of each pixel to a modulated, white neutron beam with peak energy of 3.5 Å was measured. The measured response corresponds to the changes of the pixel resistance $\Delta R$ and constitutes the signal of the pixel. Due to the difference in $T_c$, the signal from pixel 1 and 2 and the signal from pixel 3 and 4 was measured during separate, consecutive measurement series at two different operation temperatures. The operation temperatures are ${92.2}\,{\hbox {K}}$ for pixel 1 and 2 and ${91.4}\,{\hbox {K}}$ for pixel 3 and 4, and are shown in Fig. 4. The raw signal and measured reference data is shown in Fig. 8.

Figure 8a shows the measured raw signal of pixel 2 (black) and includes a slowly varying temperature background with a period of approximately ${10}\,{\hbox {min}}$. The modulation of the beam is described by the slit position (red). The opening and closing time was $t_p={30}\,{\hbox {s}}$, and the intermediate slope represents the duration of the slit movement period. The reference neutron flux measurements are shown in Fig. 8b, and includes the average integrated flux measured by the CCD reference detector for each period $t_p$ (black). The integrated flux is recorded at the end of an open or closed period. The proton current for the SINQ cold source (blue) is recorded as continuous data points and is proportional to the neutron flux.

Comparing Fig. 8a,b, the movement of the slit corresponds to the flux measured by the reference Gadox-CCD detector. The neutron beam hits the detector when the slit is open with an average flux of ${5.7\times 10^{5}}\,{\hbox {n}/{\hbox {cm}}^2/\hbox {s}}$, whereas it is mostly blocked when the slit is closed showing a baseline of ${5.1\times 10^{4}}\,{\hbox {n}/{\hbox {cm}}^2/\hbox {s}}$. The baseline is expected to be lower at the position of the bolometer detector, as this detector is placed ${30}\,{\hbox {cm}}$ behind the slit. The Gadox-CCD detector is placed ${360}\,{\hbox {cm}}$ behind the slit, and stray neutrons from the non-collimated beam is therefore expected to be counted by the reference detector that will not hit the bolometer detector.

The proton current, which is directly correlated to the neutron flux is shown in Fig. 8b. The operation of the SINQ spallation source causes short, and immediate decreases of current visible at times ${255}\,{\hbox {s}}$ and ${556}\,{\hbox {s}}$. These sudden decreases in neutron intensity can be observed in the bolometer detector signal in Fig. 8a as well as in the integrated flux of the reference detector in Fig. 8b. The patterns of the bolometer detector and source proton current match with a delay of ${1.7}\,{\hbox {s}}$ for the bolometer signal compared to the source current. Two uncertainties are significant for this delay time. As a result of the read-out sampling rate, there is an uncertainty of ${0.15}\,{\hbox {s}}$. The dominating uncertainty is caused by the time-stamp matching. The measurements of the detector and the measurements related to the beamline were performed on two separate computers using different time reference servers. Therefore, an uncertainty of $\pm {1}\,\hbox {s}$ is expected.

A slowly varying temperature background is present as a fluctuating offset between each peak of the bolometer signal visible in Fig. 8a. The thermal background fluctuations of the neutron source and surrounding temperature were measured to be approximately 100 and 5–10 times slower, respectively, than the modulated signal from the neutron source. Approximate numbers are provided as the background fluctuations were not periodic. As they are much slower and irregular, they are readily distinguishable from the input signal applying a Fourier analysis.

**Figure 9:**
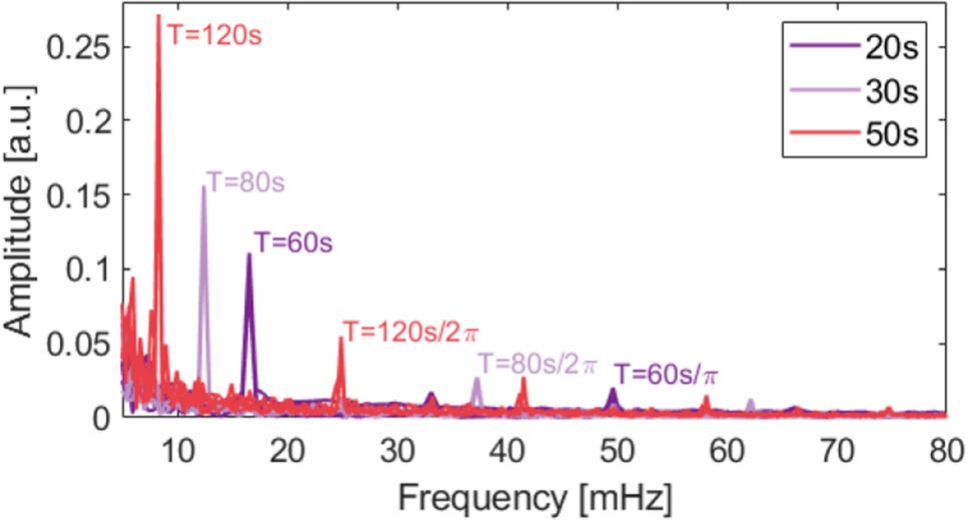
Fourier spectrum for the measured response, $\Delta R$, of all three neutron sensitive pixels. The contributions from all pixels are overlayed. The period, $T=2\cdot t_p + 2\cdot t_{{\textrm{mov}}}$, where the pulse length is $t_p=$20-${50}\,{\hbox {s}}$, and $t_{{\textrm{mov}}}={10}\,{\hbox {s}}$ is the slit movement time. Key peaks have been marked with the corresponding period. The low frequency noise $f<{5}\,{\hbox {mHz}}$ has been cut off the spectrum.

A spectrum analysis of the raw data is reported in Fig. 9. Here each series of pulse lengths ($t_p$) for all three neutron sensitive pixels (2–4) are Fourier transformed and the spectra are overlayed. The most prominent peaks and their corresponding period, *T*, are described. A period is defined as two times the pulse length, $t_p$, and two movements of the slit of approximately ${10}\,{\hbox {s}}$ each.

The spectra in Fig. 9 show a clear distinction between the modulated signals and the thermal background. The signal-to-noise ratio (SNR) is calculated for the three main peaks ($\hbox {T}={60}\,{\hbox {s}}$, ${80}\,{s}$ and ${120}\,{\hbox {s}}$) as the ratio between the power of the peak amplitude ($P_{signal}$) and the power of the amplitude of the surrounding noise floor ($P_{noise}$). The power in signal processing assumes equal resistance for the signal and noise, and thus the power reduces to $A_{signal}^2=V_{rms}^2$, where $V_{rms}$ is the root mean square of the measured voltage signal. The SNR is then calculated as,

6 $$\begin{aligned} SNR = \frac{P_{signal}}{P_{noise}}=\left( \frac{A_{signal}}{A_{noise}}\right) ^2. \end{aligned}$$

This method yields an SNR of 41, 162, and 26 for the main peaks of pulse lengths $t_p={20}\,{\hbox {s}}$, ${30}\,{\hbox {s}}$ and ${50}\,{\hbox {s}}$, respectively. For the higher order peaks of $T/2\pi$, the thermal background stabilizes, as the variations in the temperature decrease at higher frequencies. The SNR of pixels 2–4 are not significantly different. This is supported by Fig. 9, where the frequency spectra for each pixel are overlapping in the frequency range of the neutron modulation periods.

**Figure 10:**
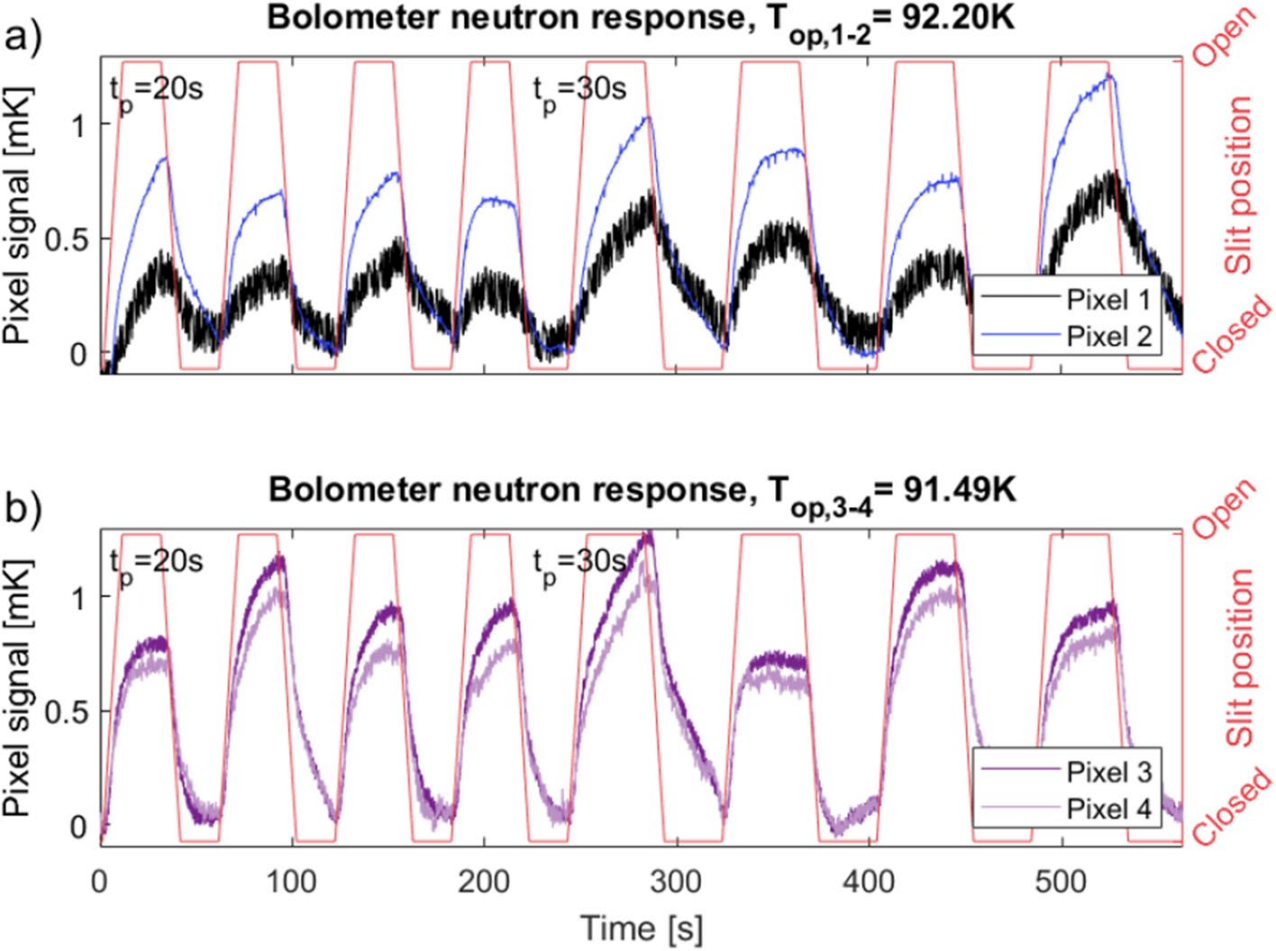
Measured detector response of pixel 1 and 2 (**a**) and pixel 3 and 4 (**b**). The varying temperature background has been subtracted, and the voltage converted to temperature using $I_{{\textrm{meas}}}$ and the *dR*/*dT*-values at the operating temperature, shown in Fig. 4. Operation temperatures of the cooler ($T_{{\textrm{op}}}$) are stated for the two measurements. The slit position (red) corresponds to the neutron beam such that en open position corresponds to a full neutron flux. The measured resistance signal for the non-neutron sensitive pixel 1 is caused by thermal cross-talk through the metallic substrate.

The thermal background has been removed by subtracting the linear regression performed between every local minimum of the signal. This is shown in Fig. 8a (dotted blue line). This method was chosen to limit the amount of artifacts for the subsequent analysis of the signal. Using a higher order filtering could have introduced artifacts, which could lead to misinterpretation of the results. This background has been subtracted for the measured signal of all four pixels shown in Fig. 10.

The measured voltage has been converted to a temperature response using the *dR*/*dT* correlation of each pixel depicted in Fig. 4. The signals reported in Fig. 10 show that the response dynamics of all pixels are similar and correspond to the neutron flux. Consistent results have been obtained for a total time of approximately 24 hours in consecutive time periods of several hours. Increased noise is observed as high frequency fluctuations for both pixel 1 ($\pm {0.1}\,{\hbox {mK}}$), and pixel 3 and 4 ($\pm {0.04}\,{\hbox {mK}}$). This is caused by an electrical noise source in the connections between the pixels and the measuring equipment.

### Thermal cross-talk

The measured response of the non-coated and thereby non-neutron sensitive pixel 1 is shown in Fig. 10a, along with the corresponding signal measured simultaneously for the neutron-sensitive pixel 2. The response of pixel 1 follows the increase and decrease of neutrons. The lack of absorbed neutrons in the transmission radiograph in Fig. 7 shows that pixel 1 is not directly sensitive to neutrons. The detector is constructed as a stack of thin films sharing a metallic substrate, as is visible in Fig. 1. Heat absorbed by the surrounding pixels, can therefore be transported through the substrate, causing thermal cross-talk. The cross-talk acts as an indirect sensitivity to neutrons.

The cross-talk for the neutron response tests is quantified by comparing the median peak height of the measured signals in pixel 1 and pixels 2–4. The peak height is defined as the difference between the local minima and maxima of each peak in a series. The average of the median pulse heights for pixels 2–4 is ${41}\,{\upmu \hbox {V}}$ for $t_p={20}\,{\hbox {s}}$ and ${45}\,{\upmu \hbox {V}}$ for $t_p={30}\,{\hbox {s}}$. For pixel 1 the median pulse heights are ${21}\,{\upmu \hbox {V}}$ and ${25}\,{\upmu \hbox {V}}$ for the two pulse lengths respectively. This is a decrease in signal for pixel 1 of 49% for $t_p={20}\,{\hbox {s}}$ and 44% for $t_p={30}\,{\hbox {s}}$. This can be observed in Fig. 10a) as the reduced height of the peaks measured for pixel 1 compared to those of pixel 2.

Previous work investigating the thermal response of this detector was performed using a laser and allowed for a single pixel to be irradiated at a time. The cross-talk signal was measured as the signal of the neighboring pixel to the one irradiated. It was found to be 50% of the full signal for pulse lengths of $t_p={30}\,{\hbox {s}}$^30^. Those values, that were previously observed by Brock et al., corresponds well with the values of 51% and 56% obtained in the present neutron response experiment.

Gamma rays are present as background at the neutron beamline and is a point of concern for most detectors. The contribution from gamma rays to the bolometer signal is expected to be small due to: (i) The BOA instrument is a very low gamma ray background beamline^25^ (a few to ${10}\,{\hbox {mSv}/\hbox {h}}$ at the entry to the beamline). This is a result of the beamline being placed at an angle from the direct line-of-sight of the neutron source. (ii) Most superconducting bolometers require a separate conversion layer to react heavily with gamma rays. This is due to the small volume of superconducting material in the pixel and the low stopping power^34^. Assuming gamma ray energies of ${10^{2}}$–${10^{5}}\,{\hbox {keV}}$^35^ the absorption of gamma rays in YBCO can be calculated using Beer–Lamberts law of attenuation:

7 $$\begin{aligned} \frac{I(z)}{I(0)}=\exp (-\mu _A/\rho \cdot z), \end{aligned}$$

where *z* is the thickness of the layer, $\rho$ is the mass density of the layer, and $\mu _A$ is the energy dependent mass attenuation constant of the material. The density of YBCO is described in Table 2, while the attenuation constants are from the NIST Standard Reference Database 126^36^. The absorption in YBCO for the low gamma ray energies of ${100}\,{\hbox {keV}}$, which have the highest absorption rates, where found to be ${1\times 10^{-4}}$. This is negligible compared to the contribution from the thermal cross-talk.

The thermal cross-talk measured in pixel 1 is expected also to be present for pixels 2–4. When up to 50% of the measured signal consists of contributions from the neighbouring pixels, each adjacent pixel can only be considered partly independent. Consequently, this cross-talk effect will be visible as a shared noise background for all of pixels 2–4. This was shown for the frequency spectra in the previous section, where the SNR values were found to be highly similar for all pixels. Indicating that all pixels share a common background visible through the substrate.

### Saturation

**Figure 11:**
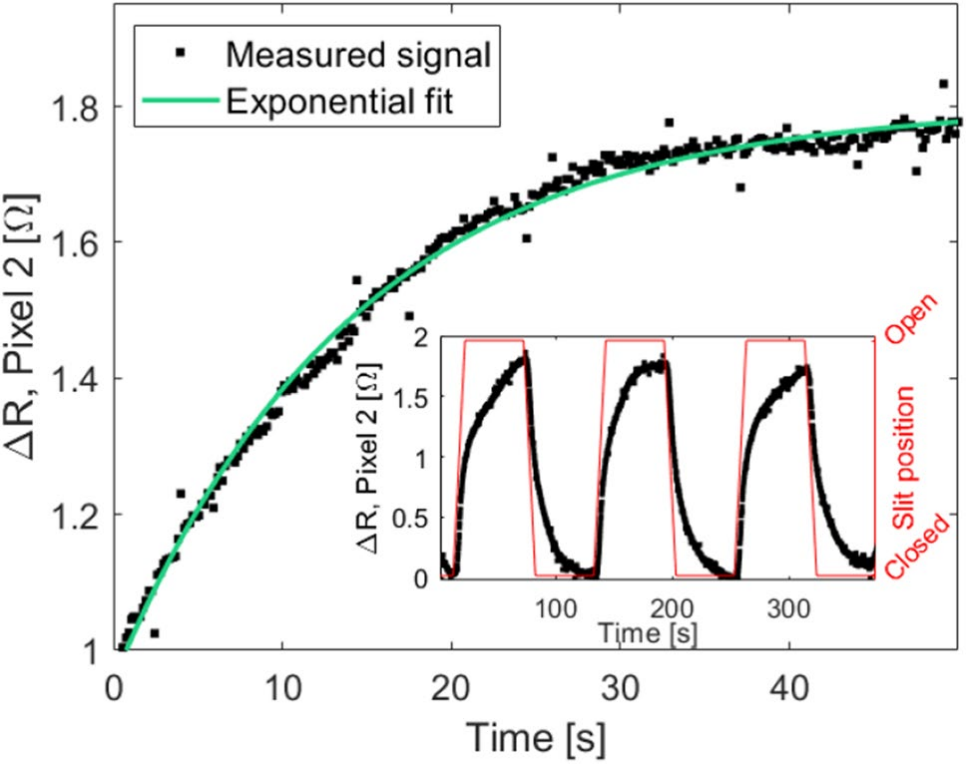
Measured voltage response (black) and an exponential fit (green) that was performed using Eq. (8). Insert shows an example of three of the fitted peaks of a $t_p={50}\,\hbox {s}$ signal with the background, *K*, removed.

The bolometer detector was tested using electronics with a sampling rate of ${8}\,{\hbox {Hz}}$. Therefore, the individual neutron pulses from the spallation source cannot be studied. When analyzing slower modulated signals, the time constants at which the detector is saturating with heat, as well as cooling to its original setpoint are important parameters. These parameters indicate if we are close to the maximum flux capabilities for the integrated flux. As is shown in the insert of Fig. 11, saturation of the test detector is slow, and changes can still be measured at the end of longer pulses with $t_p={50}\,\hbox {s}$.

The pulse shapes of the measured signals are characteristic for saturation in a circuit, where the test detector acts as a thermal capacitor in series with a thermal resistor. This is typically described with the simplified model resulting in the exponential equation reported below.

8 $$\begin{aligned} \Delta T = K+\Delta T_0\cdot \exp \left( -\frac{1}{\tau }\cdot t\right) , \end{aligned}$$

where $\Delta T$ is the total change in temperature, *K* is the temperature background level, $\Delta T_0$ is the change in temperature caused by the neutron absorption, *t* is the time variable and $\tau$ is the time constant describing the point where the signal is 1/*e* of its original value.

Equation (8) is fitted to the individual peaks in the signals measured for each of the pixels. An example of one of these fits for a period of incoming neutrons and thus increasing signal, is shown in Fig. 11. $\Delta T_0$ is found from the measured $\Delta R$ using the linear regime of the *dR*/*dT* curve shown in Fig. 4, such that $\Delta T=\Delta R\cdot (dR/dT)^{^-1}$. To avoid effects of a partial neutron signal during periods of the slit opening and closing, the data is exclusively fitted for the periods where the slit is either fully open or fully closed.

The calculated time constants for each pixel and each measurement period length, $t_p$, are found as a weighted average of the fitted parameters. Only fits with $R^2>0.95$ are used for pixels 2–4. For pixel 1 a limit of $R^2>0.75$ is used due to the increased noise of the measured signal caused by the cross-talk. The time constants can be seen in Table 1.

**Table 1:** Time constants for each pixel for each pulse length and type of signal (increasing or decreasing). The reported values on $\tau$ are the average of the constants found by fitting each individual peak of the signals of each pixel. Only fits with $R^2>0.95$ are used for pixel 2, 3 and 4, while a lower limit of $R^2>0.75$ is used for pixel 1.

$t_p$ [s]	Time constants, increasing, $\tau _{inc}$ [s]				Time constants, decreasing, $\tau _{dec}$ [s]			
	Pixel 1	Pixel 2	Pixel 3	Pixel 4	Pixel 1	Pixel 2	Pixel 3	Pixel 4
20	14 ± 5	15.1 ± 0.2	17.1 ± 0.7	19 ± 1	20 ± 6	15.2 ± 0.2	17.6 ± 0.5	18.2 ± 0.7
30	29 ± 9	16.0 ± 0.2	15.8 ± 0.3	16.9 ± 0.5	60 ± 28	21.4 ± 0.3	31.2 ± 0.9	27.7 ± 0.9
50	38 ± 6	26.1 ± 0.3	30.0 ± 0.5	30.2 ± 0.6	27 ± 2	23.4 ± 0.2	41.3 ± 0.7	33.2 ± 0.6

For pixels 2–4, the fits yield saturation time constants in an interval of $\tau _{inc}=15{-}30\,\hbox {s}$ for increasing signals and $\tau _{{\textrm{dec}}}=15{-}41\,\hbox {s}$ for the decreasing cooling phase, when the slit is closed. The time constants for pixel 1 have a much larger uncertainty due to the cross-talk as described previously. The time constants are generally larger for pixel 1, than for the remaining pixels. This is caused by the signal arriving over a larger time range as it relies on thermal transport dynamics to produce cross-talk. For pixels 2–4 this signal is directly absorbed in the neutron conversion layer deposited on top of each pixel. Therefore, the distance for the heat to be transported is shorter, and thus the transport times have a smaller variation.

**Figure 12:**
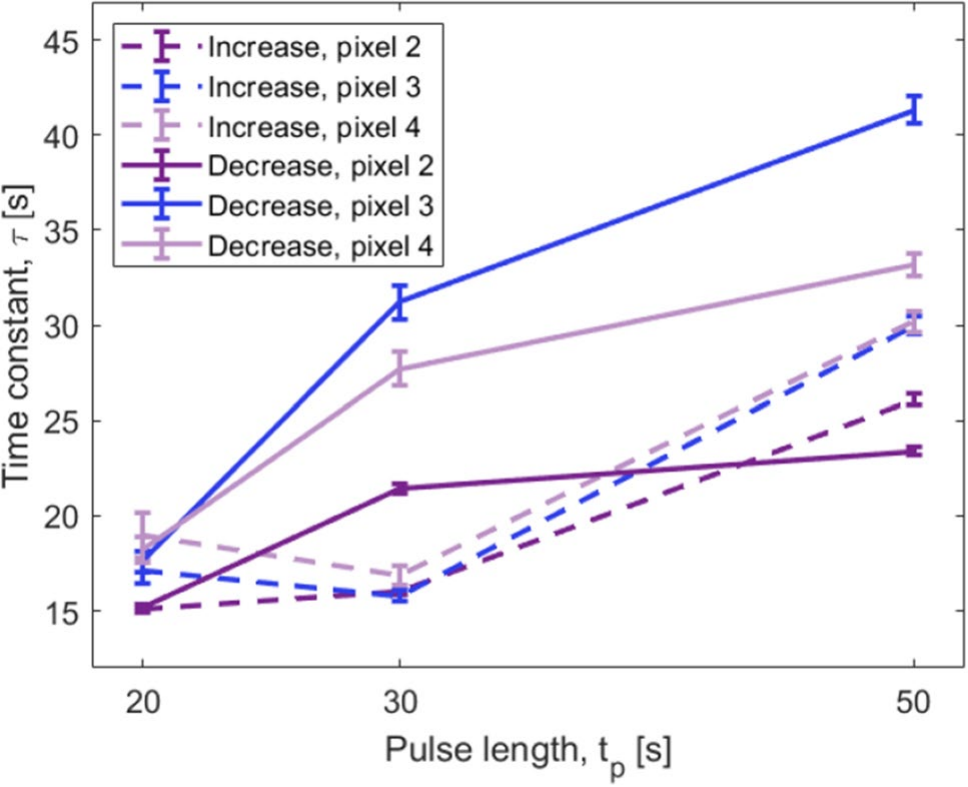
Saturation time constants, $\tau$, for each neutron-sensitive pixel and each pulse-length $t_p$. The time constant is observed to be dynamic and to follow the modulation pulse-length.

Figure 12 shows a plot of the time constants for each pixel and at each pulse length $t_p$. The three enriched boron carbide coated pixels follow the same overall trend. For the increasing signals, the 20–${30}\,{\hbox {s}}$ signal has similar $\tau$, but an increase is measured for $t_p={50}\,{\hbox {s}}$. For the decreasing signals, $\tau$ shows a gradual descent with the largest difference observed between $t_p={30}\,{\hbox {s}}$ and $t_p={20}\,{\hbox {s}}$. This indicates hysteresis in the system.

The hysteretic behaviour observed for pulse lengths of ${20}-{50}\,{\hbox {s}}$ is expected to be caused by two main contributions: (i) Previous studies of the effective specific heat of the bolometer detector have been performed using a laser with a 4–${25}\,{\hbox {Hz}}$ modulation to produce a thermal input signal. The effective heat capacity corresponded to a volume of substrate material equal to the area below the pixel and the entire thickness of the substrate. For a signal of pulse length, $t_p={30}\,{\hbox {s}}$, this effective specific heat increases to 40 times the initial value^30^. The increased, active, thermal volume leads to a slower response, which increases the saturation time constant, $\tau$, for longer pulse lengths. (ii) The detector is fastened to the top of the ceramic PCB by mechanical compression with no intermediate thermal contact layer in between. Therefore, a significant thermal resistance between the detector substrate and the PCB is expected due to the high surface roughness of the AlN ceramic. At $t_p={50}\,{\hbox {s}}$ the integrated heat increases the temperature gradient between the detector substrate and the PCB. This leads to the inclusion of the PCB in the thermal system of the detector. The thermal resistance between the substrate and the PCB results in the substrate acting as a heat reservoir, which gives rise to the observed hysteretic behavior.

For the shortest pulse length, $t_p={20}\,{\hbox {s}}$, the increasing and decreasing parts of the signal have similar time constants, $\tau _{inc}=\tau _{dec}$. This suggests that for fast measurements with rates larger than ${1}\,{\hbox {Hz}}$, the detector is cooled at the same rate as it is heated.

### Efficiency

All the neutron response tests were performed using the full energy spectrum of neutrons available at the BOA beamline. This was necessary to obtain a sufficiently high neutron flux ($\ge {10^{6}}\,{\hbox {n}/{\hbox {cm}}^{2}/\hbox {s}}$). The use of a monochromator or a collimator to obtain the efficiency of the detector at a single neutron energy would have lowered the flux significantly (up to 99%^25^) and was therefore deemed unfeasible. The spectrum has a peak neutron energy of ${6.7}\,{\hbox {meV}}$, and the full spectrum can be found in the description of the beamline^25^.

The efficiency of the test detector is calculated as the ratio of detected neutrons to the estimated number of neutrons expected to reach the detector, taking into account the geometry and beam intensity. This is described using the following expression,

9 $$\begin{aligned} \varepsilon = \varepsilon _{{\textrm{intrinsic}}} = \frac{N_{{\textrm{det}}}}{N_{{\textrm{hit}}}} = \frac{\Delta T_{{\textrm{sig}}}/(\Delta T_{{\textrm{n}}}/\textrm{d}n)}{N_{{\textrm{hit}}}}, \end{aligned}$$

where $N_{{\textrm{hit}}}$ is the number of neutrons reaching the detector, and $N_{{\textrm{det}}}$ is the number of detected neutrons. $N_{{\textrm{det}}}$ can be found as the measured change in temperature, $\Delta T_{sig}$, divided by the expected temperature increase caused by one absorbed neutron, $\Delta T_{{\textrm{n}}}/{{\textrm{d}}}n$.

The rate of neutrons hitting a single pixel ($N_{{\textrm{hit}}}$) is as,

10 $$\begin{aligned} N_{{\textrm{hit}}} = W_{{\textrm{tot}}}\cdot \alpha _{{\textrm{pix}}}\cdot (1-\alpha _{{\textrm{shield}}})\cdot A_{{\textrm{1pix}}}, \end{aligned}$$

where $W_{{\textrm{tot}}}={1.2\times 10^{8}}\,{\hbox {n}/{\hbox {cm}}^{2}/\hbox {s}}$ is the total neutron flux reaching the test detector per area. The flux is determined from an exponential fit of the gold foil measurements performed by Morgano et al.^25^. $\alpha _{{\textrm{pix}}}={20}{\%}$ is the absorption of the conversion layer measured by transmission neutron imaging as shown in Fig. 7. $\alpha _{{\textrm{shield}}}={13}{\%}$ is the absorption of the thermal shielding in the cooler environment placed between the neutron source and the detector pixels. This was measured using neutron transmission radiography similar to the method described for the individual pixel absorption ($\alpha _{{\textrm{pix}}}$). $A_{{\textrm{1pix}}}={4}\,{{\hbox {mm}}^2}$ is the area of a single pixel. The flux reaching and getting absorbed in a single pixel is found to be $N_{{\textrm{hit}}}={8.2\times 10^{5}}\,{\hbox {n}/\hbox {s}}$.

**Figure 13:**
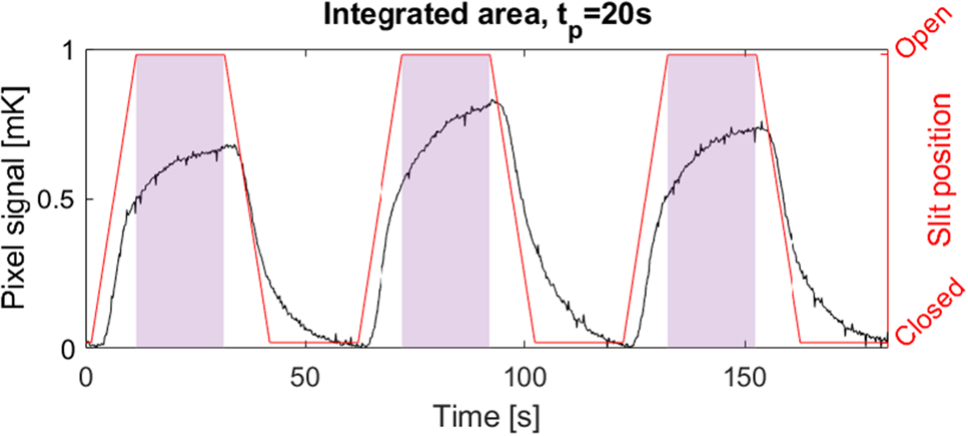
Signal with pulse length $t_p={20}\,{\hbox {s}}$ measured using pixel 2 (black). Slit movements corresponding to the incoming neutron beam (red). Integrated area used to quantify the voltage response (shaded area). The integrated area covers the period in which the slit is fully open to avoid effects of a partly blocked neutron beam.

The measured signal of the pixel is a voltage increase, $\Delta V_{{\textrm{sig}}}$, with the slowly varying temperature background, *K*, removed as previously described. The voltage signal is integrated over the entire period of opening (pulse length, $t_p$), as visualized by the shaded area in Fig. 13. This integrated voltage is then normalized with the measuring time to get a response rate.

The voltage signal can be converted to a change in resistance using the measuring current $I_{{\textrm{meas}}}={30}\,{\upmu \hbox {A}}$. The resistance is then converted to a temperature utilizing the slope of the superconducting transition (*dR*/*dT*(*T*)). The slope is determined for the operating temperature $T_{{\textrm{op}}}$ through fitting the transition curves in Fig. 4 with a logistic function. The $dR/dT(T_c)$ values are shown in Fig. 4 for the steepest points of the transitions equal to $T=T_c$. The signal is then determined as,

11 $$\begin{aligned} \Delta T_{{\textrm{sig}}}=\frac{\Delta V_{{\textrm{sig}}}}{I_{{\textrm{meas}}}\cdot dR/dT(T_c)}. \end{aligned}$$

The temperature increase expected from the absorption of a single neutron is calculated as the average over the entire pixel. Therefore, the energy of the neutrons is assumed absorbed evenly in the entire enriched boron carbide volume. This assumption is valid for the high flux regime ($>{10^{5}}\,{\hbox {n}/{\hbox {cm}}^{2}/\hbox {s}}$), in which the interaction zones of the neutrons overlap due to the high flux.

The increase in infrared radiation from the surface of the pixel caused by the increase of temperature ($\Delta T_{{\textrm{sig}}}$) was found to be an order of ${10^{-7}}$ of the deposited energy by the neutron absorption. The entire energy is therefore assumed transported downwards in the stack either to the superconducting layers (50%) or directly to the substrate(50%) due the fill-factor of 0.5. This can be described as:

12 $$\begin{aligned} \Delta T_{{\textrm{1}}}/{\textrm{d}}n=\frac{1}{2}\cdot \frac{E_{\textrm{1n}}}{A_{\textrm{SC}}\cdot t_{\textrm{SC}}\cdot \rho _{\textrm{SC}}\cdot C_{\textrm{SC}}}, \end{aligned}$$

where $E_{\textrm{1n}}={1.887}\,{\hbox {MeV}}$ is the energy deposited by a single neutron absorption event based on the analytical model described previously in this work. $A_{{\textrm{SC}}}$ and $t_{{\textrm{SC}}}$ are the area and thickness of the YBCO layer, respectively. $\rho _{{\textrm{SC}}}$ is the mass density of the YBCO layer, and $C_{{\textrm{SC}}}$ is the specific heat of YBCO at the operating temperature. The values of these parameters are listed in Table 2.

**Table 2:** Dimensions and material parameters for the superconducting YBCO layer of the detector pixels.

Symbol	Variable description	Value
$A_{{\textrm{pix}}}$	Area of entire pixel meander structure	${4}\,{{\hbox {mm}}^{2}}$
$A_{{\textrm{SC}}}$	Area of superconducting YBCO meander	${2}\,{{\hbox {mm}}^{2}}$
$t_{{\textrm{SC}}}$	Thickness of superconducting YBCO layer	${1}\,{\upmu \hbox {m}}$
$\rho _{{\textrm{SC}}}$	Density of YBCO^37^	${6370}\,{\hbox {kg}/\hbox {m}^{3}}$
$C_{{\textrm{SC}}}$	Specific heat of YBCO at 90K^38^	${189}\,{\hbox {J}/(\hbox {K}*\hbox {kg})}$

The efficiencies reported in Table 3 are obtained by combining Eqs. (9)–(12).

**Table 3:** Efficiencies calculated for each of the four pixels at different pulse lengths, $t_p$. The efficiencies for $t_p={20}\,{\hbox {s}}$ and $t_p={30}\,{\hbox {s}}$ are similar and the results were therefore pooled to gain a higher statistical significance of the result. Pixel 1 is not coated in ${^{10}{\hbox {B}}_{4}\hbox {C}}$, and the measured signal is therefore cross-talk.

$t_p$ [s]	Efficiency $\varepsilon$ [$\%$]			
	Pixel 1	Pixel 2	Pixel 3	Pixel 4
20 and 30	0.4 ± 0.2	1.4 ± 0.5	1.4 ± 0.3	1.4 ± 0.3
50	0.5 ± 0.4	1.3 ± 0.7	1.7 ± 0.6	1.7 ± 0.6

The efficiency of the enriched boron carbide coated pixels 2–4 in the prototype bolometer detector is found to be 1–2% of the absorbed neutrons. These results are about 3 times lower than the $6\%$ efficiency of a single layer with a perpendicular beam, shown for the solid-boron carbide conversion gas detectors^39^.

The efficiencies for pixels 2–4 are found to be similar within the boundaries of the calculated uncertainty. Due to the thermal cross-talk, the absorbed signal magnitude and thereby the efficiency will be shared between the pixels to some degree. Therefore, the efficiencies of pixels 2–4 can be considered as an average value for a pixel due to the physical effects of the cross-talk, rather than that of a specific pixel.

**Figure 14:**
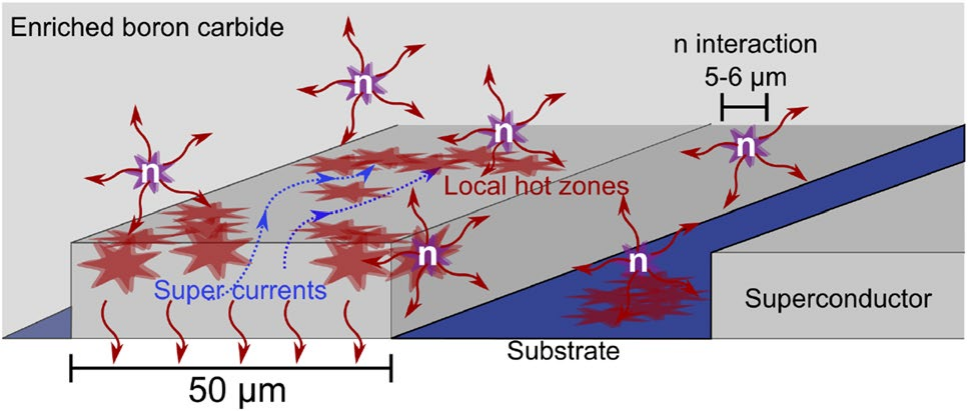
Principle sketch of the interaction areas of neutrons (red stars) absorbed near a single meander path (dark grey area). The combined mean free path of the ion products of the absorption reaction is 4–${6}\,{\upmu \hbox {m}}$, while the meander width is $w_p={50}\,{\upmu \hbox {m}}$. The supercurrents (blue arrows) have to be blocked or highly limited to produce a large signal.

The fraction of neutrons detected is directly related to the geometry of the superconductor pattern. The diameter of the interaction volume of a neutron in the conversion layer is 4–${6}\,{\upmu \hbox {m}}$. This was found by the mean free paths of the absorption products in the model section. To heat up the full width of a meander path ($w_p={50}\,{\upmu \hbox {m}}$), ten evenly distributed neutron interactions are needed at a given time. The signal in the bolometer detector is created by increasing the resistance of the superconducting path. For a signal to be measurable, the superconducting meander path needs to be fully or substantially blocked. Ten neutrons at a given time are therefore necessary to produce the highest possible signal. The mechanisms are illustrated in Fig. 14. This lower limit corresponds to a flux of ${10^{6}}\,{\hbox {n}/{\hbox {cm}}^{2}/{\hbox {s}}}$, assuming an even distribution of neutrons.

The reason that the entire meander width ($w_p$) needs to be blocked is based on the functionality of the superconducting bolometer. The transition from superconducting to normal conducting can occur both at a critical temperature ($T_c$) and at a critical current density ($J_c$). The current density is defined as $J_c=I_c/(w_p\cdot t_{sc})$, where $I_c$ is the critical current and $t_{sc}$ is the thickness of the superconductor. Both the current ($I_{{\textrm{meas}}}$) and the thickness of the superconductor is kept constant in the following discussion. Therefore, the effective width of the current carrying paths is directly correlated to the effective critical current. If the meander path is not heated all the way across $w_p$ either by direct interaction or by hot zone propagation, the super current will be transported via new pathways around the hot zones in multiple ways: (i) If these new pathways are very narrow, the measurement current might approach the critical current ($I_{{\textrm{meas}}}\approx I_c$), and the detector enters a transition phase. This will make the effective electrical resistance of the meander path increase. A signal is then detectable, although possibly reduced compared to a fully resistive transition ($I>I_c$). (ii) If the new pathways are much wider than what is needed for the transportation of the measurement current, those currents will not pass through paths with increased resistances. No output signal will then be produced.

If the width of the meander paths is reduced, a higher percentage of the absorbed neutrons will be converted to a signal. The width of the meander paths should ideally be lowered to $\le {5}\,{\upmu \hbox {m}}$. At this length-scale the lower detectable flux limit will be given by the fill factor. The present fill factor is $>{50}{\%}$, but it is possible to increase this using the currently available fabrication methods. The measurement of a single neutron interaction on a path will depend on the thermal transport properties, electronic readout, and noise.

## Discussion

An initial discussion on the possible application is presented based on the test results of this first prototype of the bolometer detector.

Several design optimizations can be implemented to increase the efficiency of this detector. One method is to minimize the path width, as described in the previous section. It would also be possible to fabricate several sensitive layers and stack them. As each ${4}\,{\upmu \hbox {m}}$ layer absorbs 20% of the incoming neutrons, a stack of 8 layers would be sufficient to absorb 95 % of the neutrons. Another option would be to operate the detector at an angle ($\theta _r$) as demonstrated by the Multi-Blade detector^23^. The increase in efficiency is proportional to $\frac{1}{\sin (\theta _r)}$^39^. For $\theta _r={5}^{\circ }$, this would lead to an increase of a factor 10. The single layer efficiency is therefore expected to be possible to increase to at least 15%, with possibilities of increasing further for smaller path geometries. All the mentioned adjustments are possible with the current methods of fabrication.

The bolometer test detector has a spatial resolution given by the pixel size of 2$\times {2}\,{{\hbox {mm}}^2}$. This is comparable to the spatial resolutions of typical neutron monitors, which are in the range of one to a few mm^40^. Fabrication of smaller pixel sizes down to hundreds of ${\upmu \hbox {m}}$ is possible, which would lead to an increased spatial resolution.

For a neutron monitor the absorption of the test detector of $20\%$ should be lowered by at least two orders of magnitude. The absorption of fast neutrons is lower than that of cold and thermal neutrons due to a lower absorption cross-section ($\sigma$) at higher velocities (*v*). This has an approximate scaling relation of $\sigma \propto 1/v$. Using the detector in a fast neutron environment (MeV) would lead to an efficiency decrease of ${10^{3}}$–${10^{5}}$. This is a suitable absorption fraction for neutron monitoring.

For thermal neutrons this could be achieved by using natural boron carbide ${^{\text{Nat}}{\hbox {B}}_{4}\hbox {C}}$ instead of enriched boron carbide ${^{10}{\hbox {B}}_{4}\hbox {C}}$. Similarly, by using a much thinner absorption layer as have been shown recently for beam monitors at the China Spallation Source^41,42^. Using a thinner enriched boron carbide absorption layer is also expected to increase the sensitivity of the pixel due to less degradation of the superconductor transition parameters as described previously^28^.

There are several advantages in the presented design for use as a high neutron flux detector or monitor. One is the current mode based read-out. The majority of neutron detectors and monitors use event-based read-out^40^, which can lead to pileup, saturation and thereby an increased dead time^43^. Measuring the flux continuously at regular intervals as a function of current is a method to avoid this problem. A further advantage is the direct readout without the need for an external amplification stage. This makes the design less sensitive to the often-observed aging effects of these electronic components in radiation environments.

## Conclusion

A high-T$_c$ superconducting Transition Edge Bolometer has been developed and tested using a neutron beam with a maximum flux of ${1.2\times 10^{8}}\,{\hbox {n}/{\hbox {cm}}^{2}/\hbox {s}}$ on the detector. The energy conversion of neutrons in the ${^{10}{\hbox {B}}_{4}\hbox {C}}$ absorption layer of the solid detector stack was modelled using an analytical model for the ion absorption. Applying the model resulted in a maximum absorption energy of ${1.877}\,{\hbox {eV}/\hbox {n}}$. The measured signal corresponds to the modulations of an incoming white neutron beam with peak energy of ${6.7}\,{\hbox {eV}}$. A significant cross-talk of up to 50% was measured between the individual pixels due to the metallic substrate. The beam modulations were performed using a slit and at a relatively low modulation frequency with periods of 60-${120}\,{\hbox {s}}$. Therefore, the temporal response was governed by a dynamic saturation curve with varying time constants corresponding to the modulation frequencies. During the ${50}\,{\hbox {s}}$ long exposures at high neutron flux, no full saturation of the detector was measured.

The efficiency was measured to be 1–2% of the absorbed neutrons for this first prototype detector. This can be optimized using the existing fabrication methods to obtain an increase of efficiency of at least an order of magnitude. Further developments in geometry of the individual pixel and the layout of the entire detector would make this detector a possible alternative to perform current-mode high flux measurements either as a detector or monitor.

## Data Availability

Contact the corresponding author for any requests regarding raw data.

## References

[1] Kirstein, O. *et al.* Neutron position sensitive detectors for the ESS. *Proceedings of Science Vertex* 8 (2014).

[2] Nagae T (2008). The J-PARC project. Nucl. Phys. A.

[3] Galambos, J. *et al.* Proton power upgrade project. Final Design Report, OAK RIDGE NATIONAL LABORATORY. ORNL/TM-2020/1570-R0; PPUP-101-TD0001-R0 (2020).

[4] Bertalot L (2019). Present status of ITER neutron diagnostics development. J. Fusion Energy.

[5] Tinguely, R. A. *et al.* Neutron diagnostics for the physics of a high-field, compact, Q $$\le$$ 1 tokamak (2019).

[6] Bertalot L (2019). Present status of ITER neutron diagnostics development. J. Fusion Energy.

[7] Mauskopf PD (2018). Transition edge sensors and kinetic inductance detectors in astronomical instruments. Publ. Astron. Soc. Pac..

[8] Esmaeil Zadeh I (2021). Superconducting nanowire single-photon detectors: A perspective on evolution, state-of-the-art, future developments, and applications. Appl. Phys. Lett..

[9] Pirro S, Mauskopf P (2017). Advances in bolometer technology for fundamental physics. Annu. Rev. Nucl. Part. Sci..

[10] Merlo V (2018). Superconducting strips: A concept in thermal neutron detection. Instruments.

[11] Ravazzani A (2006). Characterisation of the proportional counters. Radiat. Meas..

[12] Klein M, Schmidt CJ (2011). Cascade, neutron detectors for highest count rates in combination with ASIC/FPGA based readout electronics. Nucl. Instrum. Methods Phys. Res. Sect. A Accel. Spectrom. Detect. Assoc. Equip..

[13] Rhodes NJ (2012). Scintillation detectors. Neutron News.

[14] Woracek R (2019). Spatially resolved time-of-flight neutron imaging using a scintillator CMOS-camera detector with kHz time resolution. Opt. Express.

[15] Pritchard K (2021). Measuring deadtime and double-counts in a non-paralyzable scintillating neutron detector using arrival time statistics. Nucl. Instrum. Methods Phys. Res. Sect. A Accel. Spectrom. Detect. Assoc. Equip..

[16] Miyajima S (2016). Development of a neutron imager based on superconducting detectors. Physica C Supercond. Appl..

[17] Shishido H (2018). High-speed neutron imaging using a current-biased delay-line detector of kinetic inductance. Phys. Rev. Appl..

[18] Iizawa, Y. *et al.* Energy-resolved neutron imaging with high spatial resolution using a superconducting delay-line kinetic inductance detector. *Supercond. Sci. Technol.* **32** (2019). 10.1088/1361-6668/ab4e5c. arXiv:1911.02374.

[19] Höglund C (2012). B4C thin films for neutron detection. J. Appl. Phys..

[20] Piscitelli, F. *Boron-10 layers, Neutron Reflectometry and Thermal Neutron Gaseous Detectors*. PhD thesis, Università degli Studi di Perugia; Institut Laue-Langevin (2013). arXiv:1406.3133

[21] Piscitelli F (2014). Study of a high spatial resolution10b-based thermal neutron detector for application in neutron reflectometry: The multi-blade prototype. J. Instrum..

[22] Anastasopoulos M (2017). Multi-grid detector for neutron spectroscopy: Results obtained on time-of-flight spectrometer CNCS. J. Instrum..

[23] Piscitelli F (2018). Characterization of the multi-blade 10b-based detector at the crisp reflectometer at ISIS for neutron reflectometry at ess. J. Instrum..

[24] Maulerová, V. *et al.* First evaluation of a novel ionisation chamber for thermal neutron beam monitoring. *Epj Tech. Instrum.* **9** (2022). 10.1140/epjti/s40485-022-00086-x

[25] Morgano M, Peetermans S, Lehmann E, Panzner T, Filges U (2014). Neutron imaging options at the boa beamline at Paul Scherrer Institut. Nucl. Instrum. Methods Phys. Res. Sec. A Accel. Spectrom. Detect. Assoc. Equip..

[26] Blau B (2009). The swiss spallation neutron source SINQ at Paul Scherrer Institut. Neutron News.

[27] Atikur Rahman, M. A review on cuprate based superconducting materials including characteristics and applications. *Am. J. Phys. Appl.* **3**, 39. 10.11648/j.ajpa.20150302.15 (2015).

[28] Brock MB (2022). Strain effects of absorbing layer on superconducting properties of a high-flux neutron detector. IEEE Trans. Appl. Supercond..

[29] Convert P, Forsyth J, Laue-Langevin I (1983). Position-sensitive Detection of Thermal Neutrons.

[30] Brock MB, Østergaard EV, Wulff AC, Abrahamsen AB, Kuhn LT (2023). Effective thermal properties of a HTS transition edge bolometer for high-flux neutron detection. Adv. Electron. Mater..

[31] Buyuk B, Tugrul AB (2014). Gamma and neutron attenuation behaviours of boron carbide-silicon carbide composites. Ann. Nucl. Energy.

[32] Lundstedt C, Harken A, Day E, Robertson B, Adenwalla S (2006). Modeling solid-state boron carbide low energy neutron detectors. Nucl. Instrum. Methods Phys. Res. Sect. A Accel. Spectrom. Detect. Assoc. Equip..

[33] Ziegler JF, Ziegler MD, Biersack JP (2010). SRIM—The stopping and range of ions in matter (2010). Nucl. Instrum. Methods Phys. Res. Sect. B Beam Interact. Mater. Atoms.

[34] Terracol, S. F. *et al.* Ultra-high resolution gamma-ray spectrometer development for nuclear attribution and non-proliferation applications. *2004 IEEE Nuclear Science Symposium Conference Record (IEEE Cat. No. 04ch37604)* 1006-13, vol. 2 (2004).

[35] Miceli A (2014). Measurements of gamma-ray background spectra at spallation neutron source beamlines. J. Anal. Atom. Spectrom..

[36] Hubbell, J. H. & Seltzer, S. M. Nist standard reference database 126. *Nucl. Instrum. Methods Phys. Res. Sect. A Accel. Spectrom. Detect. Assoc. Equip.* **NISTIR 5632**. 10.18434/T4D01F (2004).

[37] Knizhnik A, Shter GE, Grader GS, Reisner GM, Eckstein Y (2003). Interrelation of preparation conditions, morphology, chemical reactivity and homogeneity of ceramic YBCO. Physica C Supercond. Appl..

[38] Pavese F, Malishev V (1994). Routine measurements of specific-heat capacity and thermal-conductivity of high-TC superconducting materials in the range 4–300-K using modular equipment. Adv. Cryog. Eng..

[39] Piscitelli F, Van Esch P (2013). Analytical modeling of thin film neutron converters and its application to thermal neutron gas detectors. J. Instrum..

[40] Issa F (2017). Characterization of thermal neutron beam monitors. Phys. Rev. Accel. Beams.

[41] Liu L, Yu Y, Yu M, Zhang Z, Yang Y (2023). Realization of a thin boron layer neutron beam monitor. Nucl. Instrum. Methods Phys. Res. Sect. A Accel. Spectrom. Detect. Assoc. Equip..

[42] Zhu L (2023). A ceramic-gem neutron detector with a wide neutron-flux measurement range for the beam monitoring at china spallation neutron source. J. Instrum..

[43] Tavernier S (2010). Experimental Techniques in Nuclear and Particle Physics.

